# SART and Individual Trial Mistake Thresholds: Predictive Model for Mobility Decline

**DOI:** 10.3390/geriatrics6030085

**Published:** 2021-08-31

**Authors:** Rossella Rizzo, Silvin Paul Knight, James R. C. Davis, Louise Newman, Eoin Duggan, Rose Anne Kenny, Roman Romero-Ortuno

**Affiliations:** 1 The Irish Longitudinal Study on Ageing, Trinity College Dublin, D02 R590 Dublin, Ireland; silvin.knight@tcd.ie (S.P.K.); davisj5@tcd.ie (J.R.C.D.); louise.newman@tcd.ie (L.N.); dugganeo@tcd.ie (E.D.); rkenny@tcd.ie (R.A.K.); romeroor@tcd.ie (R.R.-O.); 2 Discipline of Medical Gerontology, School of Medicine, Trinity College Dublin, D02 PN40 Dublin, Ireland; 3 Mercer’s Institute for Successful Ageing, St. James’s Hospital, D08 NHY1 Dublin, Ireland; 4 Global Brain Health Institute, Trinity College Dublin, D02 PN40 Dublin, Ireland

**Keywords:** sustained attention to response task, SART, multimodal visualization, threshold, timed up-and-go, falls, cognition, repeated measures, mobility decline

## Abstract

The Sustained Attention to Response Task (SART) has been used to measure neurocognitive functions in older adults. However, simplified average features of this complex dataset may result in loss of primary information and fail to express associations between test performance and clinically meaningful outcomes. Here, we describe a new method to visualise individual trial (raw) information obtained from the SART test, vis-à-vis age, and groups based on mobility status in a large population-based study of ageing in Ireland. A thresholding method, based on the individual trial number of mistakes, was employed to better visualise poorer SART performances, and was statistically validated with binary logistic regression models to predict mobility and cognitive decline after 4 years. Raw SART data were available for 4864 participants aged 50 years and over at baseline. The novel visualisation-derived feature *bad performance*, indicating the number of SART trials with at least 4 mistakes, was the most significant predictor of mobility decline expressed by the transition from Timed Up-and-Go (TUG) < 12 to TUG ≥ 12 s (OR = 1.29; 95% CI 1.14–1.46; *p* < 0.001), and the only significant predictor of new falls (OR = 1.11; 95% CI 1.03–1.21; *p* = 0.011), in models adjusted for multiple covariates. However, no SART-related variables resulted significant for the risk of cognitive decline, expressed by a decrease of ≥2 points in the Mini-Mental State Examination (MMSE) score. This novel multimodal visualisation could help clinicians easily develop clinical hypotheses. A threshold approach to the evaluation of SART performance in older adults may better identify subjects at higher risk of future mobility decline.

## 1. Introduction

Computer-based neurocognitive tests are commonly utilised in research [1], and increasingly, in clinical practice, for both the detection and rehabilitation of cognitive disorders in adults [2]. However, the raw outputs from computer-based tests pose methodological and interpretation challenges, owing to the lack of optimal assays for the precise characterisation of latent neurocognitive processes, and shortcomings of many current methods to allow direct visualisation of multi-modal data that could help clinicians generate more meaningful hypotheses and predictions [3,4]. These challenges are only magnified in the case of computer-based repeated-measures neurocognitive data stemming from large-scale studies.

A common approach in many research designs has been to simplify the raw computer outputs into average features, such as mean and standard deviation of response time, as surrogates of overall performance and variability [5]. However, by simplifying the data in this fashion, much primary information is lost and, consequently, researchers can be left facing unexpected lack of association between results from this approach and clinical outcomes of interest (e.g., disease severity categories). In this scenario, researchers have suspected that the loss of physiologically important information in the simplified data analysis may be responsible for the inability to detect clinically expected associations [5]. Indeed, information averaging can result in loss of power and once a simplified predictor has been created and used in analyses, results should be interpreted considering the dimension of the derived variable, not at the level of the individual original variable [6].

The Sustained Attention to Response Task (SART) is a standard computer-based cognitive test designed to measure the sustained attention, a fundamental executive function for completing tasks that require supervision over time [7]. Sustained attention is a result of the interaction between two different subsystems: vigilance and arousal (alertness) [8,9]. Vigilant attention allows to detect subtle changes in the environment occurring over long periods of time [8,10], and relies on a network of cortical areas including the cingulate gyrus, prefrontal cortex and inferior parietal lobule, as imaging studies have demonstrated [11,12]. The maintenance of an adequate level of arousal is necessary to detect target stimuli [8]. Electrophysiology and functional neuroimaging studies have demonstrated that arousal is activated through a subcortical network including the thalamus and noradrenergic brainstem structures [13,14]. The SART is a continuous performance reaction-time (RT) task designed to promote attention lapses; participants are required to monitor visual displays acknowledging responses for frequent neutral signals (GO trials), but withholding response when detecting rare targets (NO–GO trials) [5,15]. Commission errors (responding to NO–GO trials) or omission errors (failure to respond to GO trials) reflect lack of vigilance, while the RT is a measure of alertness. In older adults, SART has been shown to be correlated with frailty [16] and falls efficacy [17]. However, due to its complex granular intrinsic structure, the optimal way to approach the analysis of SART data remains the subject of debate.

Moreover, recent studies [18,19] have shown a complex network of interactions among different physiological systems and, particularly, between the brain and the locomotor system. We hypothesised that having information on the cognitive and mobility status in the same SART data visualisation would provide clinicians with a more comprehensive framework of general physiological status, even in the absence of clear clinical evidence of mobility or cognitive disorders, and therefore help formulate hypotheses related to potential future health risks.

The Timed Up-and-Go (TUG) is a well-established test to measure mobility and predict risk of falls in older adults [20,21]. In the framework of longitudinal studies of ageing, there is not a unique consensus on the relationship between mobility and cognition. Recent works have shown that baseline quantitative gait parameters are significant predictors of cognitive decline and dementia in older adults [22,23]. However, a previous study in community-dwelling older adults who were cognitively intact at baseline demonstrated the absence of associations between baseline mobility and future cognitive decline, where the latter was expressed by traditionally derived SART variables and other cognitive measures [24].

On the other direction of the association, recent studies have suggested that older participants with poorer choice reaction or stop-signal reaction times may display an accelerated pattern of mobility decline [25] and have a higher risk of incident falls [26]. As this type of study had not previously been attempted with SART data, we were therefore motivated to investigate potential relationships between SART performance and the risk of either a clinically meaningful mobility decline or future falls, utilising novel approaches to classify participants based on their baseline SART performance. Moreover, we considered the Mini-Mental State Examination (MMSE) score as a standard measure of overall cognitive status [27], and in line with previous works, we also investigated the hypothetical association between SART performance and a clinically meaningful decline in MMSE score.

To address the above hypotheses more effectively through the utilisation of the raw SART data, we aimed to devise a new method to visualise the full information obtained from the SART tests performed by a large sample of older participants in a large population-based study, which allowed us to extract features otherwise potentially hidden in derived variables. The individual trial (raw) data visualisation allowed ordering by continuous variables (e.g., age) and also discrete groups of clinical interest (e.g., baseline mobility impairment present versus absent). Furthermore, we formulated a new thresholding method based on the individual trial percentage of mistakes to individuate a subset of participants considered to have a poor SART performance, tested the correlations of this new subdivision with mobility decline measured by TUG and gait speed, risk of future falls, and cognitive decline, and compared its predictive power with other traditional global SART parameters.

## 2. Materials and Methods

### 2.1. Dataset

#### 2.1.1. Design and Setting

This research was carried out as part of The Irish Longitudinal Study on Ageing (TILDA), an ongoing nationally representative prospective cohort study of community-dwelling adults. TILDA collects information on the health, economic, and social circumstances of people aged 50 years and over in Ireland. Participants were randomly recruited based on their geographic location. The full design of the study and cohort characteristics have been described elsewhere [28,29]. Wave 1 of the study (baseline) took place between October 2009 and February 2011 and included (i) a comprehensive health assessment conducted at a dedicated health assessment centre (HAC) and (ii) a computer-assisted personal interview (CAPI) in participants’ own homes, which involved a collection of detailed data on health, social, and economic factors. Wave 3 of TILDA was conducted between March 2014 and December 2015 (approximately 4 years after wave 1) and comprised the same modes of data collection described above. Ethical approvals for each wave were granted from the Health Sciences Research Ethics Committee at Trinity College Dublin, Dublin, Ireland, and all participants provided written informed consent. All research was performed in accordance with the Declaration of Helsinki.

#### 2.1.2. SART Protocol

The SART is a computerised continuous performance reaction time (RT) task [7]. It requires participants to respond to a repeating stream of consecutive digits 1 to 9 (GO trials) but withhold responding to the digit 3 (NO–GO trials).

In the SART test, each digit appears for 300 milliseconds (ms), with an interval of 800 ms between digits. The cycle of digits 1 to 9 is repeated 23 times, giving a total of 207 trials. The test lasts for approximately 4 min. Participants are instructed to press a keyboard key as soon as possible (with RT automatically recorded using Presentation version 16.5) for each digit presented. RT is null (RT = 0) for the appearance of the digit 3 when no mistakes are committed. For a hypothetical perfect task, there are 8 × 23 = 184 non-null values corresponding to the RT when the participant is supposed to press the key, and 23 null values corresponding to the trials when the participant is not supposed to press the key. In practice, over the course of the test, many participants lose attention and commit mistakes. Two types of mistakes can be detected in the data: commission errors (i.e., responding to NO–GO trials), which reflect lapses of sustained attention; and omission errors (i.e., failure to respond to GO trials), reflecting a break from task engagement, also corresponding to lapsing attention [5]. In this work, we considered SART data from wave 1 of TILDA.

#### 2.1.3. Mobility Variables

- *TUG*: TUG measures the time (seconds) taken for a participant to stand up, walk 3 m at normal pace along a line on the floor, turn around, walk back to the chair, and sit down [20]. The test is not just a measure of physical ability, in fact it requires an individual to process instructions, plan and execute movements, focusing on the task and avoiding distractions. This cognitive component makes the test more complex than straight-line walking. Generally, a cut-off of 12 [21,30] or 14 [31,32] seconds (s) is clinically used to discriminate participants with significant mobility impairment. The TUG in wave 1 ($TUG_{1}$) and wave 3 ($TUG_{3}$) were utilised in this study. Given our aim to capture risk of early mobility decline in this relatively healthy community-based sample, we chose the more restrictive cut-off of 12 s to define clinically significant mobility impairment in both waves. Specifically, we defined mobility decline (*TUG decline*) for a given participant when $TUG_{1}$ was less than 12 s ($TUG_{1}<12$) and $TUG_{3}$ larger or equal than 12 s ($TUG_{3}\geq 12$).
- *Gait speed*: gait speed was assessed using a computerised walkway (4.88 m GAITRite™ (CIR Systems Inc., Franklin, NJ, USA) pressure sensing mat) [24,33]. Participants performed two walks at usual pace, starting and finishing 2.5 m before and 2.0 m after the walkway. The measured gait speed was calculated as an average between the two walks and did not include the acceleration and deceleration phases. Cut-offs between 30 and 120 cm per second (cm/s) are generally used to individuate mobility disability (range 30–100 cm/s) [33] and slow usual pace in older adults (range 80–120 cm/s) [34,35,36]. We considered the usual gait speed (UGS) at wave 1 ($UGS_{1}$) and at wave 3 ($UGS_{3}$), and defined *UGS decline* for a given participant when $UGS_{1}$ was greater or equal than 100 cm/s ($UGS_{1}\geq 100\mathrm{cm}/s$) and $UGS_{3}$ smaller than 100 cm/s ($UGS_{3}<100\mathrm{cm}/s$).
- *Falls*: as part of the CAPI, participants were asked whether they had falls in the year prior to the interview. We recorded the number of recalled falls in wave 1 ($falls_{1}$) and wave 3 ($falls_{3}$), and defined as *new fallers* those participants who had at least 1 fall in the year prior to the examination at wave 3 ($falls_{3}>0$) and no falls in the year prior to the examination at wave 1 ($falls_{1}=0$).

#### 2.1.4. MMSE

Global cognitive function was assessed using the Mini-Mental State Examination (MMSE) test, giving participants a score from 0 (minimum) to 30 (maximum) [27,37,38]. We considered the MMSE score in wave 1 ($MMSE_{1}$) and wave 3 ($MMSE_{3})$ and, in line with previous recommendations [39], defined as clinically meaningful cognitive decline a decrease of at least 2 points between wave 1 and 3 ($MMSE_{1}\;-\;MMSE_{3}\geq 2$).

#### 2.1.5. Covariates

Several potentially relevant covariates at wave 1 were considered in this work: (a) features extracted from the SART multimodal visualisation (see below), in addition to the traditional SART mean and standard deviation (SD) of RTs (across all trials) both measured in milliseconds (ms); (b) sociodemographic variables: age, sex, and education level (categorised as primary/none, secondary or third/higher); (c) variables expressing the psychological status of participants: anxiety, assessed with the anxiety subscale of the Hospital Anxiety and Depression Scale (HADS-A) [40], which ranges in scores from 0 to 21 (higher scores indicating more symptoms of anxiety); depression, assessed with the Centre for Epidemiological Studies Depression (CES-D) scale [41], which ranges in scores from 0 to 60 (higher scores indicating worse depressive status); and (d) variables related to the physical status of participants: whether or not they were taking any antihypertensive medications (coded using the Anatomical Therapeutic Chemical Classification (ATC) [42]: antihypertensive medications (ATC C02), diuretics (ATC C03), β-blockers (ATC C07), calcium channel blockers (ATC C08), and renin-angiotensin system agents (ATC C09)), had history of diabetes, self-reported smoking (categorised as never, past, or current) and alcohol consumption habits (the answer to the question “Do you have a drinking problem?” (yes, no, or I don’t know) was recorded), UGS at baseline, and physical activity status based on the International Physical Activity Questionnaire (IPAQ) (short form) scoring protocol [43] (categorised as low, medium, or high).

### 2.2. Multimodal Visualisation

All analyses and graphical representations were created with MATLAB (R2020b, The MathWorks, Inc., Natick, MA, USA).

#### 2.2.1. Entire Sample

1. *SART RT representation*: we considered the number of mistakes (commission and/or omission errors) committed within each trial, and the average RT for all correct actions in that trial. We then represented a spot for each trial and participant, the position of which depended on its average RT, and the size on the percentage of errors committed in the corresponding trial. Thus, each participant had 23 spots arranged on the same vertical line, corresponding to the 23 SART trials. SART performance is known to be influenced by age [44]; therefore, we recorded the age of participants and ordered the visualisations by increasing age as a continuous variable. The spots corresponding to different participants were organised horizontally from youngest (left) to oldest (right). For ease of interpretation, ticks were created to indicate a 5-year age change: the distances on the horizontal axis between two consecutive ticks, corresponding to a 5-year range, were not always the same, since most of the cohort was between 50 and 65 years old. Of note, trials where the participant did not press the key at any time and/or some RT were missing, did not have the corresponding computed average RT, and were not plotted in the graph. Bigger spots correspond to trials with higher number of mistakes. Moreover, the spots were also colour-coded based on the percentage of mistakes, going from light brown (0 mistakes) to black for the maximum number of mistakes (8 mistakes in order to have at least 1 RT and assign the position to the spot). Spots with size larger than 2 SD from the mean size calculated across all trials and all participants (excluding missing data and trials with 0 correct actions) were highlighted by white edges in the graph and labelled as “big spots”. A complete mathematical explanation of the SART RT representation is given in an appendix to this work (Appendix A).
2. *SART mistakes line*: to further visualise our dataset, we calculated the sum of mistakes made by each participant across all trials, and we represented this value as an additional line function in the same graph above the previously explained cloud plot. The mistakes line function is not linearly related to the size of the spots and indicates a global parameter for each participant over the whole task.
3. *MMSE and TUG lines*: additionally, the graph was enriched by the presentation of participants’ MMSE score and TUG at wave 1. These values were multiplied by a factor 3 to be visible at the same graph scale.

#### 2.2.2. Thresholded Multimodal Visualisation

To ease the visual detection of “big spots” and highlight poor performances that may have been ‘buried’ in the dense cloud plot, a second graph was created containing only the “big spots”. All of the above-mentioned notations regarding the coordinates, size and colour of the spots still apply. The curves representing the number of mistakes, MMSE score, and TUG were now limited to only subjects who had at least one big spot.

#### 2.2.3. Application to Categories Stratification and Threshold

We applied the above-described multimodal visualisation methods to allow additional categorisation to discern participants with $TUG_{1}\geq 12$ s. Specifically, SART trials for participants with low TUG were presented with dark blue spots, and the trials for participants with high TUG with light blue spots on the right part of the graph, sequentially after the low TUG participants along the horizontal axis. Notably, we ordered participants by age within each category. Therefore, we could appreciate in the same graph multiple levels of information of the dataset analysed: (1) SART RT characteristics across different trials for each participant, and across different participants; (2) distribution of the SART RT by age and for different baseline TUG categories; and (3) global parameters like number of total mistakes, MMSE score, and TUG represented as continuous curves in the top part of the graph (where void spots in the curves were due to missing data for some participants).

#### 2.2.4. Feature Extraction

We extracted some features directly from the multimodal visualisation: (a) mean RT and SD RT, (b) whether or not a participant had bad performances (“big spots”), especially in the thresholded visualisation, and (c) based on the size and colour of the spots, it was possible to understand how each participant performed in each trial, and if their performances were consistent to each other in the whole task in terms of mistakes committed, or if the variation of performance between different trials was high. We subsequently created a new variable that indicated the number of bad performances for each participant (i.e., number of big spots).

### 2.3. Statistical Analysis

We considered the evolution of mobility variables over time. Specifically, we compared the distribution of TUG and UGS values in the same group of participants at wave 1 and wave 3 using the Wilcoxon test, a nonparametric test used to compare related samples [45,46]. We then compared the TUG and UGS change between participants who at wave 1 did not have any SART bad performances, and those who had at least one bad performance. We compared these two subsets of participants using the Mann–Whitney U test, a nonparametric test used to compare independent samples [47,48]. All the statistical tests were performed in IBM SPSS Statistics version 27 (IBM Corp., Armonk, NY, USA). Statistical significance was set at p<0.05 throughout.

#### 2.3.1. Binary Logistic Regression

Binary logistic regression models were used to predict the binary outcomes that we considered to be clinically meaningful. Specifically, we tested whether the new variable reporting the number of “bad performances” in SART at wave 1 was a good predictor of mobility decline: we assigned 1 to participants with *TUG decline* as defined in Section 2.1.3, and 0 otherwise; correspondingly we assigned 1 to participants with *UGS decline* as defined in Section 2.1.3, and 0 otherwise. Similarly, we assigned 1 to *new fallers*, as defined in Section 2.1.3, and 0 otherwise. Moreover, we also tested the prediction strength of the new variable for cognitive decline, as defined in Section 2.1.4. These 4 dichotomous variables were set as outcomes in the binary logistic regression models, from which we reported the odds ratio (OR) with corresponding 95% confidence interval (C.I.) and *p*-value for each independent variable in the model. The OR expresses the odds that an outcome will occur in the presence of an independent variable, compared to the odds that the outcome will occur in the absence of that variable, therefore if OR>1 the independent variable influences positively the odds of the outcome, if OR<1 the independent variable influences negatively the odds of the outcome, i.e., it is “protective” against the outcome, and if OR=1 the independent variable does not influence the outcome [49,50]. For each binary outcome, wave 1 covariates were used in four different regression models to incrementally determine the robustness of the predictor, as follows: model 1, with just the predictor; model 2, which was model 1 additionally adjusted with mean RT and SD RT; model 3, which was model 2 with the addition of age, sex, and education level; and model 4, which was the fully adjusted regression model, considering also all the other covariates mentioned in Section 2.1.5 (anxiety, depression, hypertensives, diabetes, smoking, alcohol, and IPAQ).

#### 2.3.2. Comparison with Two Other Potential Predictors

In order to test the prediction strength of our new SART feature of interest (i.e., ‘number of bad performances’) for the four binary outcomes of clinical interest, the same four different binary logistic regression models were applied considering the same covariates mentioned before but substituting the ‘number of bad performances’ with the global variable ‘number of total mistakes’ in the whole SART task, and the ‘number of mistakes in good performances’. The latter was obtained summing up the number of mistakes in performances that did not reach the threshold defined in Section 2.2.1, and therefore did not amount to “big spots”. Of note, every time that we applied the binary logistic regression model (whether adjusted by covariates or not) we considered only one of these three potential predictors, because we were interested to individuate a variable that had good predictive power for the outcome and could be used independently from the other predictors. Indeed, the presence of different predictors would lead to a mixed effect on the outcome probability, and the predictive power would depend on the combination of predictors, and not on the individual predictors. Each adjusted model, considering the three different predictors separately, had been tested for multi-collinearity (based on Spearman’s correlation). We compared the OR of the three predictors, whilst noting the degree of overlap in the 95% C.I.s and the corresponding *p*-values.

#### 2.3.3. Sensitivity Analysis

In order to test the robustness of the new variable *bad performances*, and to evaluate its ability to predict the outcomes in the binary logistic regression models compared to the performance of the other two SART-related predictors, we considered a further logistic model, model 4a, which was model 4 adjusted with a mobility-related covariate UGS at baseline wave 1. In this case, we considered the significance of the three main potential predictors, and compared their OR, whilst noting the degree of overlap in the 95% C.I.s.

## 3. Results

In total, 8175 participants over the age of 50 years were included in wave 1 of TILDA, of which 5035 attended the health centre assessment. Among those, SART data were available for 4864 participants (54.6% female, aged 50 to 93 years, with mean 61.7 ± 8.3 years). Table 1 presents descriptive statistics for the variables used in this work for the baseline wave 1 cohort (N=4864), and the merged cohort for waves 1 and 3 (N=3890).

### 3.1. Information Provided by the Multimodal Visualisation

**Figure 1:**
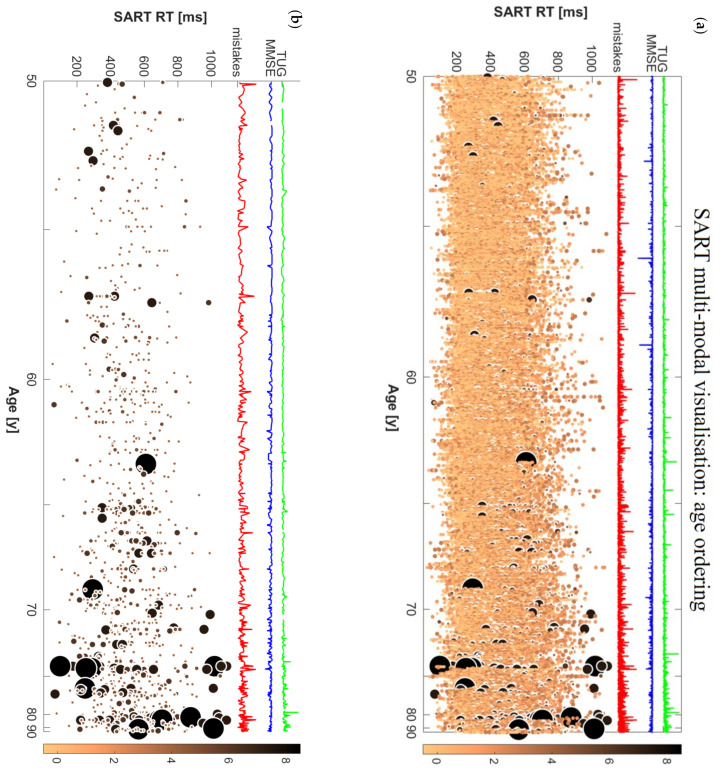
Multimodal visualisation of SART data, (**a**) ordered by age and (**b**) ordered by age with thresholding applied.

Figure 1 shows the multimodal visualizations based on N=4864 participants. There were in total 1222 “big spots” representing bad performances for 565 different subjects (11.6% of the sample). Among those aged 50–64, 8.2% had bad performances; among those aged 65–74, 17.9% had bad performances; and among those aged 75 years and older, 33.7% had bad performances. In this dataset, the “big spot” (bad performance) threshold, as defined in Section 2.2.1, was 4 mistakes out of 9 for each individual trial. The density distribution of big spots can be better appreciated in Figure 1b.

**Figure 2:**
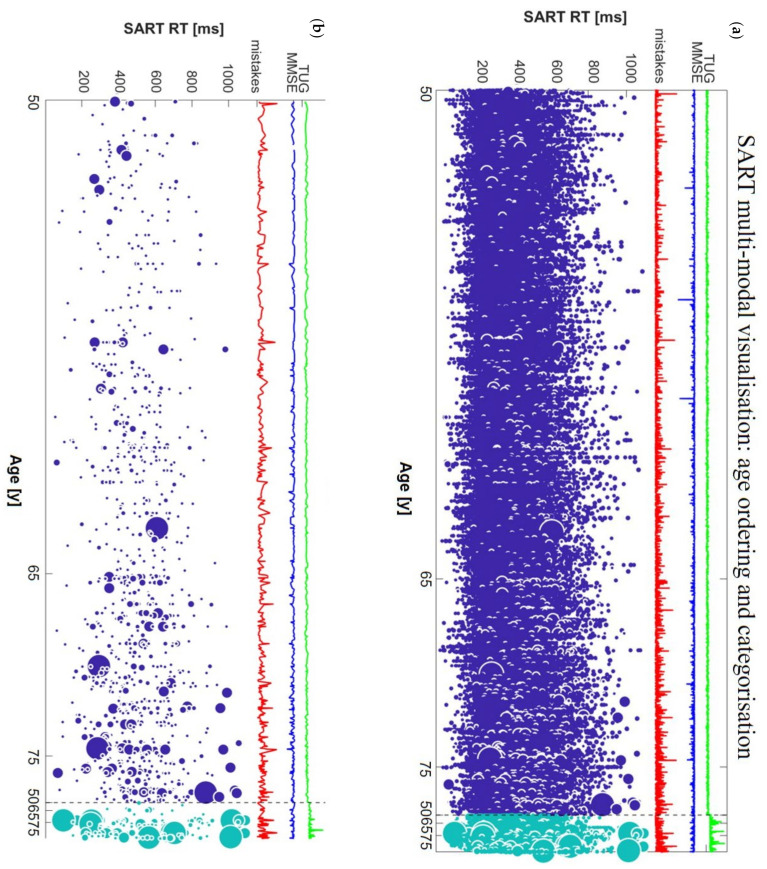
Multimodal visualisation of SART data, (**a**) ordered by age within baseline TUG categories and (**b**) ordered by age within TUG categories with thresholding applied. Dark blue spots indicate participants with $TUG_{1}<12$ s, light blue spots indicate participants with $TUG_{1}\geq 12$ s.

From N=4864, 4834 had baseline TUG information (data for this variable was missing for 30 participants, i.e., 0.6% of the entire sample); among these, 237 participants had $TUG_{1}\geq 12$ s. Figure 2 shows the multimodal visualisation discriminating participants with low $TUG_{1}$ from those with high $TUG_{1}$. Moreover, within each category, the subjects are age-sorted in ascending order. Considering the participants with bad performances, we registered that 29.1% of participants with $TUG_{1}\geq 12$ s had at least 1 SART bad performance, while only 10.8% of participants with $TUG_{1}<12$ s had SART bad performances.

Regarding missing data, in both visualisations there were 83 subjects whose corresponding spots could not be depicted in the graphs. Among those, 20 subjects had 1 or 2 trials where they did not press the key at all having not even 1 RT in that trial. In the rest, there was missing data just for some RT; because of this, it was not possible to calculate the average RT for that trial, and the corresponding spot in the graph could not be created.

### 3.2. Longitudinal Analysis

The merged longitudinal sample examined at both waves 1 and 3 was constituted by N=3890 participants (54.6% female, ages 50 to 90 years, with mean 61.5 ± 8.1 years). Table 1 shows additional characteristics of this sample. We compared the distributions of *TUG*, *UGS* and *previous falls* at waves 1 and 3, and the Wilcoxon rank sum test suggested that the distributions of the three variables were significantly different: *p* < 0.001 for *TUG* and *UGS*, and *p* = 0.015 for *falls*.

**Figure 3:**
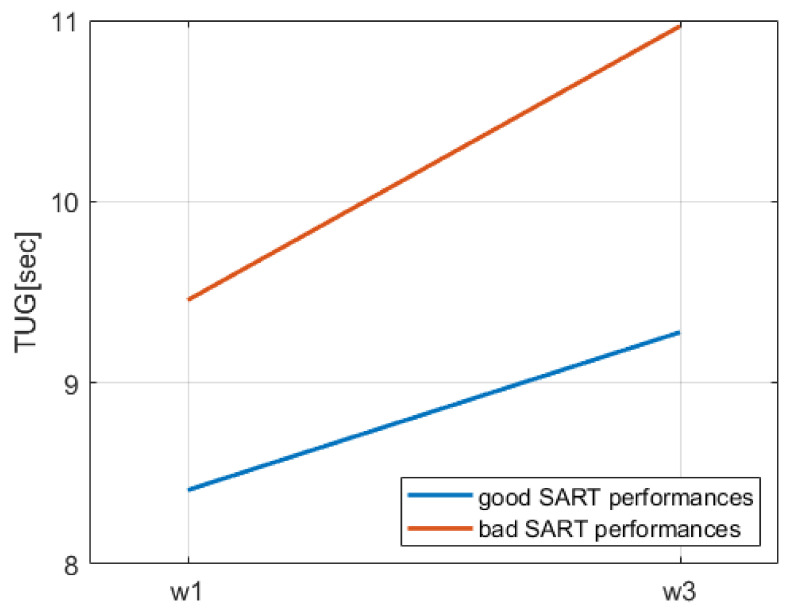
TUG at wave 1 and wave 3 for participants who only had good performances in SART at wave 1 and those who had at least 1 bad performance.

Figure 3 shows the mean TUG at wave 1 and 3 for two subgroups of participants: one where participants only had good performances in SART at wave 1, and another where participants had at least one bad performance. From Figure 3, we noticed two elements: (i) TUG generally increased from wave 1 to wave 3; and (ii) the increment of TUG between the two waves seemed more pronounced in participants who had at least one SART bad performance at wave 1. Indeed, the slope of the TUG increment for participants with only good performances was m = 0.871, while the slope for participants with at least one SART bad performance was m = 1.512. The distributions of values for $TUG_{3}-\;TUG_{1}$ were statistically significantly different between the two subgroups (Mann–Whitney U test *p* < 0.001).

Furthermore, significant differences were also found between the distributions of values for $UGS_{1}-\;UGS_{3}$ and the number of previous falls at wave 3 for participants with only good SART performances at wave 1 and participants with at least 1 bad performance (Mann–Whitney U test *p* = 0.014 for UGS decrease and *p* = 0.016 for falls).

### 3.3. Number of Bad Performances as Predictor of Mobility Decline

The three potential SART predictors, *bad performances*, *total mistakes* and *mistakes in good performances*, failed the Kolmogorov–Smirnov and Shapiro–Wilk normality tests (p<0.001, i.e., their distributions were not significantly similar to the normal distribution). We also noted that the standardised residuals were not normally distributed. Therefore, we excluded the linear regression model and any other parametric tests and applied binary logistic regression models for the prediction of the four dichotomous outcomes of clinical interest. In every model, the independent variables passed the multi-collinearity test (Spearman’s correlation coefficient $\left|\rho \right|\;\leq \;0.422$ for all pairs) and satisfied all other logistic regression assumptions.

As Table 2 shows, the binary logistic regression models demonstrated that the number of bad performances was a significant independent predictor of *TUG decline* (*p* < 0.001 in all four models, OR = 1.287, 95% C.I. = (1.137; 1.456) in the fully adjusted model (model 4), i.e., for every one-unit increase in *bad performances* we would expect an increase of 0.287 in the odds of *TUG decline*, and OR = 1.305, 95% C.I. = (1.130; 1.508) in model 4a). Table A1 in Appendix A shows the results of the fully adjusted binary logistic regression model 4 where the OR, 95% C.I. for OR and *p*-value for each independent variable in the model are reported. Of note, other significant predictors of *TUG decline* in model 4 were age, being on antihypertensives, history of diabetes, and active smoking status. A high level of self-reported physical activity was significantly protective against *TUG decline*, i.e., those who were highly physically active were less likely to have a TUG≥12 s after 4 years. We observe that in model 4a, where UGS at baseline was also considered as a covariate, the variable *bad performances* maintained its statistical significance, while other covariates, which were significant predictors in the previous model 4, lost it (Table A2 in Appendix A). In this case the only significant predictors were the number of bad performances, age and UGS at baseline. Moreover, comparing the OR of *bad performances* across different models applied, we noted that it was stronger in model 1, it decreased in models 2 and 3, and it increased again in model 4, and even more in model 4a, having a difference of just 0.083 compared to model 1.

The variable *bad performances* was a significant predictor for *UGS decline* only in model 1 (*p* < 0.001, OR = 1.232, 95% C.I. = (1.102; 1.377)), namely without considering other covariates. In models 2, 3, and 4, the number of bad performances was not a significant predictor (*p* > 0.05). Table A3 in Appendix A shows the results of the fully adjusted model 4 where the OR, 95% C.I. for OR, and *p*-value for each independent variable in the model are reported. In this case the only significant independent predictors were age and being on antihypertensive medications.

Furthermore, we applied the same binary logistic regression models for the prediction of becoming a new faller. As shown in Table 3, in models 1, 2, 4, and 4a the *bad performances* feature resulted a significant predictor (*p* < 0.021 in the four models, OR = 1.114, 95% C.I. = (1.026; 1.211) in the fully adjusted Model 4, and OR = 1.110, 95% C.I. = (1.021; 1.207) in model 4a), while in model 3 its *p*-value was borderline (p=0.057), having the absolute value of the difference from the significance threshold α=0.05 smaller than $10^{-2}(\left|p-\alpha \right|<0.01$). Table A4 in Appendix A shows the results of the fully adjusted binary logistic regression model 4 reporting the OR, 95% C.I. for OR and *p*-value for each independent variable. Of note, the only other significant predictor of becoming a new faller was age (*p* = 0.004, OR = 1.020, 95% C.I. = (1.006; 1.034)). Table A5 in Appendix A presents similar results for model 4a: again the only other significant predictor of becoming a new faller was age (*p* = 0.011, OR = 1.019, 95% C.I. = (1.004; 1.034)). Moreover, we noted that the OR of *bad performances*, although decreased in models 2 and 3, in model 4 it assumed the same value of that in model 1, and slightly different in model 4a (overlapping 95% C.I.).

### 3.4. Comparison with Other Potential Predictors

Table 2 shows a comparison of the OR, reporting also the 95% C.I. and *p*-value, for the three predictors in the five different logistic regression models, as defined in Section 2.3.1. In each model, all predictors were significantly associated with the outcome *TUG decline*. However, the variable *bad performances* always had a larger OR than that of other predictors, and without overlap of 95% C.I.s, suggesting its larger weight in the prediction of this outcome.

Table A6 in Appendix A shows the results for the prediction of the other variable expressing mobility decline, *UGS decline*, in all four binary logistic regression models for each of the three main potential predictors: *bad performances*, *total mistakes and mistakes in good performances*. In models 3 and 4, none of the three predictors were significantly associated with the *UGS decline*, while *bad performances* was significant in model 1, and *total mistakes* and *mistakes in good performances* were significant in models 1 and 2. We note that in model 1 *bad performances* had the highest OR with non-overlapping 95% C.I. compared to *total mistakes* and *mistakes in good performances.*

We repeated the procedure for the outcome *new faller*, and the results of the comparison are shown in Table 3. Again, the independent variable *bad performances* performed better than the other two predictors in model 1, having a larger OR and a non-overlapping C.I. In model 3, the *p*-value for *bad performances* was borderline $(\left|p-0.05\right|<0.01$), while for *total mistakes* and *mistakes in good performances* it was statistically insignificant (p≫0.05). In models 4 and 4a, *bad performances* emerged as the only variable that was statistically significant for the prediction of *new faller*, with OR = 1.114 in model 4, i.e., for every one-unit increase in *bad performances* we would expect an increase of 0.114 in the odds of becoming a new faller, and OR = 1.110 in model 4a.

Our findings suggested that the independent variable *bad performances* was more predictive of mobility decline and risk of new falls than the other two candidate variables derived from the SART visualisation, i.e., *total mistakes* and *mistakes in good performances*.

### 3.5. Absence of Association with Cognitive Decline

As shown in Table 4, the variable *bad performances* was not a significant predictor of *MMSE decline* (*p*-value = 0.187) in the fully adjusted model 4, nor in model 4a. *Total mistakes* and *mistakes in good performances* were not significant predictors either in the fully adjusted models. Table A7 in Appendix A shows the results of the binary logistic regression model 4 fully adjusted per covariates considering the independent variable *bad performances* and having as outcome *MMSE decline*. In this case, the only significant predictors were age (*p* < 0.001, OR = 1.044, 95% C.I. = (1.029; 1.059)) and current smoking (*p* = 0.013, OR = 1.523, 95% C.I. = (1.092; 2.123)), both positively associated with increased odds of *MMSE decline*. Table A8 in Appendix A presents similar results for model 4a, with the only difference that beside age (*p* < 0.001, OR = 1.041, 95% C.I. = (1.025; 1.057)) and current smoking (*p* = 0.020, OR = 1.491, 95% C.I. = (1.066; 2.085)), third or higher education level was significant for the outcome, specifically protective against an *MMSE decline* (*p* = 0.043, OR = 0.738, 95% C.I. = (0.549; 0.991).

Table 4 shows the comparison between *bad performances*, *total mistakes*, and *mistakes in good performances* in the prediction of *MMSE decline*, reporting OR, and corresponding 95% C.I and *p*-value for the five binary logistic regression models employed. Of note, all three predictors were significant for *MMSE decline* in model 1 (i.e., where each of the three predictors was the only independent variable in the model), and in this case *bad performances* had the highest OR with non-overlapping 95% C.I. compared to *total mistakes* and *mistakes in good performances*.

## 4. Discussion

### 4.1. Multimodal Visualisation

In the present study, we devised a new methodology for the multimodal visualisation of big repeated-measures data with continuous variable ordering and categorical stratification, and we exemplified this with the case of raw SART performance data, accompanied by MMSE and TUG values, sorted by age, and stratified by baseline TUG performance.

By using this novel type of visualisation, clinicians could gain a deeper understanding as to how a complex repeated-measures dataset is articulated across different subjects and across repeated measures (SART trials in this case). Moreover, using the ordering of a continuous variable (age in this case) in the whole dataset (Figure 1) and within each category (Figure 2) allows one to compare the performance of different subjects by age and to formulate hypotheses that can then be tested with formal statistical analyses. Furthermore, by using the threshold for bad performance (Figure 1b and Figure 2b) one can more clearly visualise their distribution across subjects and RTs. This could be the first step in the formulation of a new model to assign cognitive scores to different individuals based on the co-existence of a wide range of different types of parameters.

The visualisations helped us quickly appreciate that bad performances were rare in younger participants (i.e., in their 50s) and concentrated around lower RTs, while in older subjects (i.e., in their 70s and 80s), a wider distribution of bad performances was suggested across a wider range of RTs. By using the thresholding visualisation method, we were able to gain a more focused insight into the participants who had bad SART performances and cross-inspect them with corresponding global parameters of clinical interest such as the total number of SART mistakes, MMSE, and TUG values.

Indeed, the most important characteristic of our visualisation method is the possibility to rapidly visualise raw data and gain immediate insights as to the possible correlations with different kinds of parameters. This multimodal raw data inspection can help visually identify anomalies and outliers in the data, in a way that is diluted and often undetected in traditional designs based on average measures. For example, a high peak in the total mistakes line can be due to one bad performance or multiple performances with just 1 or 2 mistakes, which, in our case would not be labelled as “bad performance” since our threshold required 4 mistakes for the definition of a “bad performance”. The superimposed MMSE and TUG curves further underscore multi-modality by providing a global cognitive and mobility score for each participant. The possibility to look at them together with the whole distribution of SART RT values (not just derivative global variables for SART) can provide a more nuanced understanding of the combined cognitive and mobility status of an individual.

Raw data visualisation can therefore support the generation of multiple novel hypotheses involving the relationship between test performance features and other modalities of clinical interest. For example, in our visualisations it was clear that longer TUG times corresponded to higher concentrations of bad performances, and even to the biggest spots among bad performances, i.e., those with very high number of mistakes (biggest light blue spots in Figure 2b). Moreover, we could notice that ‘dips’ in MMSE seemed to have a very modest association with SART RT performance [51]. Not only were the lowest MMSE scores generally not in correspondence with the high number of total SART mistakes, they were not even present among participants with bad performances, as can be seen comparing panels (a) and (b) in Figure 2. Therefore, we directed our interest towards investigating possible associations between SART bad performances at wave 1 and risk of mobility and/or cognitive decline at wave 3 after 4 years.

### 4.2. Individual Trial Mistake Threshold in Longitudinal Analysis–Mobility Decline

The cross-sectional considerations on possible associations among SART performance, mobility, and cognitive status, which emerged from the multimodal visualisation, were explored in this study at a longitudinal level. A great advantage of the TILDA study is that it allows for the investigation of variation in certain variables over time, thanks to data being collected longitudinally across different waves [16,21,25].

The longitudinal power of the TILDA study has been used in many recent works aiming to understand correlations between different physiological systems and formulate hypotheses on the possible prediction of mobility and/or cognitive decline [21,24,25]. However, the precise mechanisms governing the longitudinal relationship between cognitive and mobility status are still unclear.

Gait disorders and mobility impairment are very common in older adults [34,52], and are often related to neurological diseases [53,54]. Current literature suggests the presence of correlations between cognitive and motor function in older adults [25,55]; specifically, it has been shown that gait abnormalities could precede and predict the onset of cognitive decline [56,57]. Various standard measures of cognitive status have been used in recent studies, usually in the form of derived variables that while giving a simplified insight on complex repeated-measures data, could carry the risk of losing relevant primary information [5,24]. Recent findings have demonstrated that baseline mobility, expressed by gait parameters and TUG were not significant predictors of cognitive decline in community-dwelling older adults who were cognitively intact at baseline [24]. However, other recent works [22,23] have shown significant associations between baseline quantitative gait parameters and risk of cognitive decline and dementia. Investigating the aforementioned correlations in the opposite direction, recent longitudinal studies [25] suggested that longer motor response time in a choice reaction test could be a significant predictor of accelerated mobility decline, although this effect was statistically and clinically small.

Furthermore, recent studies have shown associations between variability in SART and risk of falls and falls efficacy [17]. Falls are very common amongst older persons [58,59], affecting them not only in the moment of the fall itself, but also later with irreversible consequences, especially in people living with higher levels of frailty [60,61]. Consequences may not only be physical, but also psychological, since some fallers often voluntarily reduce their movements after falling fearing to fall again, and this eventually leads to deconditioning and weakness that in turn increase the risk of further falls [62].

In our study, we aimed to introduce not only a visualisation that would shed light on the whole information contained in a complex dataset like SART, but also individuate a subset of participants containing key information to predict mobility decline and risk of falls in older adults. We considered the outliers for 2 SD from the mean of the distribution of the number of mistakes committed across the different SART trials and across all participants. Such outliers’ trials were labelled as “bad performances” if the participant committed at least four mistakes out of nine possible correct actions. The new thresholding method individuated a new variable expressing the number of bad performances for each participant. We noted that only 565 participants had at least 1 bad performance, compared to the whole cohort at wave 1 of 4864 participants. Therefore, the subset defined by the threshold was only the 11.6% of the entire dataset, and we hypothesised that the defined subset could contain valuable information to predict the risk of mobility decline.

We considered the temporal evolution of mobility status, expressed by TUG, UGS, and history of falls at waves 1 and 3, and found that not only the distributions of *TUG*/*UGS*/*falls* at the two waves were statistically significantly different from each other, but significant differences were also found for longitudinal TUG increment $\left(TUG_{3}\;-\;TUG_{1}\right)$ and longitudinal UGS decrease ($UGS_{1}-UGS_{3}$) between the subgroup of participants with only good SART performances at wave 1 and participants with at least one SART bad performance. Further investigating this, we found that our new SART variable *bad performances* was a significant predictor of *TUG decline* in the employed binary logistic regression models, being associated with an increase per unit of around 30% in the odds of having TUG decline in the fully adjusted models. Moreover, and consistently with previous literature on cardiovascular burden and mobility limitations in older adults [63], we noted that in model 4, advancing age, the presence of antihypertensives and diabetes, and current smoking status were significant positive predictors of *TUG decline*, bringing an increase in the probability for the outcome of about 14%, 94%, 68%, and 79%, respectively. Moreover, in keeping with the literature [64] and clinical expectation, a significant negative predictor of *TUG decline* was a high level of self-reported physical activity, which decreased by 32% the probability of *TUG decline*. However, considering also UGS at baseline as a covariate in model 4a we noted that some independent variables lost significance in the prediction of *TUG decline*; the only significant positive predictors were *bad performances*, advancing age, and antihypertensive medication use, which determined an increase of 30%, 10%, and 67%, respectively, on the probability of the outcome, while higher UGS at wave 1 was protective against *TUG decline*, leading to a decrease of 6% in the probability. We note that the difference of results between models 4 and 4a were probably due to the high correlation between $UGS_{1}$ and $TUG_{1}$, and between $UGS_{3}$ and $TUG_{3}$ (Spearman’s correlation coefficient $\left|\rho \right|\geq 0.700$ at the significance level of 0.01). Therefore, it was highly probable that UGS at baseline would influence the probability of having a TUG decline after 4 years. Nevertheless, we note that even considering a variable strongly associated with the outcome, *bad performances* did not lose its significance, demonstrating it to be a robust predictor of *TUG decline*.

Our findings suggested that participants with SART bad performances and with a normal TUG at wave 1 ($TUG_{1}<12$ s) had a 30% greater probability to have a TUG at wave 3 indicating a mobility impairment (i.e., $TUG_{3}\geq 12$ s). Moreover, comparing the contribution of *bad performances* in the five models employed, we noticed that (i) even adding covariates, it remained a significant predictor, suggesting its robustness in the prediction of the outcome, and (ii) although its OR decreased in models 2 and 3 compared to model 1, it increased again in model 4, and even more in model 4a. The latter observation suggests that in models 2 and 3, other covariates significantly influenced the probability of the outcome, however these variables were not robust for the model, since the presence of further covariates in model 4 and 4a made their presence not significant for the model. In this case, *bad performances* remained significant and regained part of the prediction power temporarily lost in model 3. Furthermore, considering model 4a, where a variable (*UGS*) highly correlated to the outcome was used as a covariate, *bad performances* not only did not lose significance, but also its prediction power increased further, taking part of the weight from less robust independent variables, which were significant predictors in model 4.

Equivalently, we employed the first four logistic regression models for the prediction of *UGS decline*. In this case, *bad performances* was a significant predictor only in model 1, but was not significant in the fully adjusted model. To explain the difference in the results between *TUG decline* and *UGS decline*, we need to understand how these two mobility measures were taken. To measure UGS, participants were required to simply walk in a straight line. This task, then, does not require any major cognitive involvement, since walking is an action that is normally executed automatically in independent adults. Differently, TUG task requires participants to stand up, walk in a straight line, come back, and sit again. Thus, this test is more cognitively involved than straight-line walking, as the individual needs to process and remember instructions, plan and execute movements, focus on the task, and avoid distractions [20]. SART *bad performances* could capture cognitive processes that are similar to those required for completion of the TUG, and this could be a possible explanation as to why *bad performances* independently predicted future mobility decline in our analyses.

Similarly, we considered SART *bad performances* as one of the independent variables in binary logistic regression models for the prediction of becoming a new faller at wave 3. We found that our new variable was a significant positive predictor in the fully adjusted models. In fact, the presence of SART *bad performances* in participants who did not have any falls at wave 1 contributed to an 11% additional probability of falls at wave 3, compared to those who did not have any SART bad performances at wave 1, i.e., who never hit the threshold of 4 mistakes in one trial. We noted that among all the other covariates used in the model, only age was a significant predictor of becoming a new faller at wave 3, although with a low positive contribution of only 2% to the odds of the outcome. Even in this case, comparing the *bad performances* contribution in the five models employed, we observed a phenomenon similar to that for the prediction of *TUG decline*. Indeed, we could notice a decrease in its prediction power in models 2 and 3 with additional loss of significance in model 3 due to the presence of significant predictors among the added covariates. However, in model 4 and 4a it reacquired significance and predictive weight, suggesting the non-robustness of previously significant covariates.

#### Comparison with Traditional SART Measures as Predictors

Traditional SART variables measure global features, such as the total number of mistakes (omission and/or commission errors) in the whole task, and the mean RT and SD RT across the whole task [5,17,20,24,44]. However, using global parameters, which average a large complex dataset, such as the SART, important information residing in individual trials in the set of repeated measures may be lost. Indeed, no significant associations between SART global parameters and mobility status had been previously found [17,24]; and even when correlations involving reaction time measures had been found, the statistical effect was quite small [25].

In the present study, we aimed to define a new variable, which, being more selective, could discriminate the participants with greater risk of mobility decline. We demonstrated that our variable *bad performances* was a significant predictor of risk of TUG decline and becoming a new faller. Furthermore, we compared its predictive power with other potential predictors: the global parameter *total mistakes* and *mistakes in good performances*, obtained by summing up all the mistakes in SART good performances, i.e., where the maximum number of mistakes per trial was less than 4. We noted that the variables *bad performances* and *mistakes in good performances* were almost complementary, because *mistakes in good performances* considers all the mistakes that are not reaching the threshold for the definition of a bad performance.

In the literature, there is not a uniform consensus on the method to follow in order to compare the importance of different predictors in binary logistic regression [21,65,66]. We compared the models with the different potential predictors for *TUG decline*, *UGS decline*, and *new fallers* considering the OR with non-overlapping 95% C.I. when the independent variable was significant for the prediction of the outcome.

In the fully adjusted models for the prediction of *TUG decline*, we found that, although all three variables considered were significant as predictors, the new variable *bad performances* had a higher OR compared to *total mistakes* and *mistakes in good performances*, where the difference between ORs considering the C.I. was equal or greater than 0.092 in model 4, and 0.083 in model 4a. Specifically, while the effect of the variable *bad performances* per unit was around 30% on the probability of the outcome, *total mistakes*, and *mistakes in good performances* had an effect per unit of only 3% on the probability of the outcome, namely 1 magnitude less than *bad performances*.

Regarding the prediction of *UGS decline*, no SART-related variables were significant as predictors for the outcome in the fully adjusted model. *Bad performances*, *total mistakes,* and *mistakes in good performances* were all significant in model 1, where *bad performances* assumed the highest OR, and *total mistakes* and *mistakes in good performances* were also significant in model 2, but they all lost their significance in models adjusted for covariates. This result may be due to the fact that, while UGS is a simpler measure of physical mobility, the TUG task is more cognitively involved and, thus, logistic regression models were able to detect the correlation with SART variables.

Moreover, we found that *bad performances* significantly predicted *new falls*, i.e., falls at wave 3 for participants who did not have any falls at wave 1, while *total mistakes* and *mistakes in good performances* were not significant predictors. Namely, our findings suggested that for participants who did not report any falls at wave 1, the number of mistakes in SART task was not a risk of falls at wave 3, as long as they did not hit the threshold of 4 mistakes in a single trial.

Furthermore, comparing the three predictors’ performance across the five models employed, we noticed that in the prediction of both *TUG decline* and risk of becoming a new faller, the predictors’ *total mistakes* and *mistakes in good performances* did not manifest the same phenomenon observed for *bad performances*. Namely, the presence of covariates in models 2 and 3 was associated with a decrease in prediction power, expressed by the OR, of *total mistakes* and *mistakes in good performances* for both outcomes, with additional loss of significance in model 3 for *total mistakes* and models 2 and 3 for *mistakes in good performances* in the prediction of *new fallers*. Differently from the models involving *bad performances*, in this case in model 4 and 4a the predictors did not reacquire prediction power nor significance for the prediction of *new fallers*, suggesting that they were not robust and strong enough in the prediction, and possibly other covariates revealed to be significant in the model.

Our results suggest that when SART mistakes reach threshold status, the number of times that this happens should be taken seriously as potentially heralding mobility decline and/or falls; however, mistakes below threshold level were less predictive and this could be used to reassure participants that ‘one swallow does not make a spring’ when it comes to interpreting the clinical significance of a participant making sub-threshold mistakes during the SART task. This still agrees with the principle that clinicians who administer tests of neurocognitive performance (such as the SART) should be reluctant to attribute poor test performance to anxiety that occurs during the testing process [67], but at the same time argues in favour of not placing undue emphasis on the clinical significance of mistakes that occur below a proven threshold. Interestingly, anxiety was not a significant covariate in any of the fully adjusted logistic regression models, which further dilutes the potential mechanistic role of anxiety in the prediction of the four clinical outcomes under study. Another interesting insight from our analyses is that once the focus was on the new visualisation features and additional covariates, mean SART RT and SD of RT had no independent effect on the prediction of any of the outcomes. Since RT variables have been the main focus of previous research on SART-related health outcomes, we would support the need to revisit those studies fort the potential effects of thresholded error features as reported herein.

### 4.3. Individual Trial Mistake Threshold in Longitudinal Analysis–Cognitive Decline

SART and MMSE are two standard tests used to evaluate cognitive functions: the first specifically measures the sustained attention, the ability to be vigilant over time, and the second gives a more global score on the cognitive status [27]. Cross-sectional studies have suggested only modest associations between SART performance, considering traditional measures, and MMSE score [51,68]. In our study, we investigated the predictive ability of our new variable SART *bad performances* and other independent variables *total mistakes* and *mistakes in good performances* for cognitive decline, expressed by a decline on MMSE score of at least 2 points after 4 years. We found that all three variables considered were not significant predictors of *MMSE decline* in the fully adjusted models. We note that, similarly to previous works [24], a decline in MMSE score was very hard to detect at wave 3 in our sample for different reasons: (i) the participants attending the TILDA health assessment centre (where the SART test was administered) were relatively high-functioning community-dwelling adults with good cognitive and physical health [69]; (ii) the MMSE test performed at each wave has always the same structure, and therefore participants can show learning effects potentially resulting in even higher MMSE scores over time and absence of general decline [70,71]. To overcome this potential limitation, future work could investigate these correlations over a longer time period, or with different global cognitive tests.

### 4.4. Strengths and Limitations of the Study

One of the main strengths of our study is the large dataset and comprehensive health assessment; indeed, TILDA is one of the most detailed population-based longitudinal studies of ageing, and the comprehensive measures and tests taken at different waves constitute the main strength for longitudinal analyses involving various physiological systems. Specifically, the complex SART dataset offers the possibility of investigating repeated measures for a large sample of individuals. As discussed above, the multimodal visualisation carried out in the present study allows to easily observe the large sample organised by age, stratified by categories of interest, and allow visual associations with other measures included in the health assessment. The new threshold feature based on SART single trials, is not only easy to determine by clinicians, but also offers a meaningful measure to identify subjects at risk of mobility decline over a 4-year period. As done with other types of data [72], this novel visualisation methodology could form the basis of a web-based platform able to facilitate each of the mentioned processes by integrating the different phases into an intuitive system with a graphical user interface that hides the complexity underlying each of the data modalities used, and presents the results in a flexible and visual way, avoiding any manual handling of data during the process.

Our study also has potential limitations, for example in the individuation of outliers in the distribution of single trial mistakes across all participants. Many studies [73,74] prefer to remove outliers present in a distribution, because this is not considered to be a good representation of the sample population. However, our analyses demonstrated that, although the subset of participants individuated by the new threshold was relatively small compared to the entire sample, it could serve for the prediction of mobility decline with a good statistical power, and even better than traditional global parameters usually used to characterize the mistakes in SART performances. Another limitation is that the analytical wave 1 sample was only based in TILDA participants who attended the health assessment centre and, as such, findings cannot be considered as necessarily representative of the entire Irish community-dwelling population. In this regard, given the “healthy participant effect” associated with attendance to the health assessment centre [69], together with the 4-year attrition effect, it is possible that our findings may have somehow underestimated the ability of the new SART features to predict mobility decline, new falls, and even cognitive decline. It would be therefore important to attempt to replicate this study in frailer cohorts in the future.

## 5. Conclusions

In conclusion, the multimodal visualisation carried out in the present study allowed us (i) to appreciate the richness present in the complex raw SART data, which can be otherwise lost using derivative variables; (ii) to rapidly visually inspect a large amount of data; and (iii) inspect the dataset together with different health variables of clinical interest in order to generate hypotheses. In this representation, we were able to look at the entire dataset and compare the participants’ performances (between each other) by age; we were also able to correlate the single performance in each trial with global parameters such as number of total mistakes, MMSE and TUG, aiming to have a comprehensive visual overview of the cognitive and physical status of each subject. Based on the visualisation, a newly defined mistakes threshold for individual SART trials was statistically validated. The determined subset presented higher risk of future mobility decline, as measured by *TUG decline*, and falls with a larger statistical effect than other candidate measures, and could therefore be used to identify early signs of health disorders and prioritise individualised clinical interventions to lower the risks. A threshold approach to the evaluation of SART performance in older adults may better identify subjects at higher risk of future mobility decline and/or falls.

## Figures and Tables

**Table 1:** Descriptive statistics for the whole set of variables considered in this study at wave 1 for the entire dataset (cohort 1, N=4864 ) and the merged dataset (cohort 2, *N* = 3890). The first part of the table gives minimum and maximum values, and mean and SD for each continuous variable. The second part shows ordinal or nominal variables and their frequency in percentage.

Continuous Variable	Cohort 1 (Wave 1): Mean (SD); Range	Cohort 2 (Merged Wave 1–3): Mean (SD); Range
SART bad performances	0.3 (1.1);	0.2 (1.0);
	0–21	0–20
SART: Total mistakes	11.0 (12.3);	10.3 (11.7);
	0–117	0–117
SART: Mistakes in good performances	9.8 (9.6);	9.3 (9.1);
	0–60	0–60
SART: Mean RT (ms)	385.3 (96.1);	383.3 (94.4);
	168.9–836.5	168.9–836.5
SART: SD RT (ms)	72.9 (41.6);	71.3 (40.6);
	12.8–364.2	12.8–364.2
TUG (s)	8.6 (2.1);	8.5 (1.9);
	4.3–39.3	4.8–28.7
UGS (cm/s)	136.0 (20.5);	136.6 (19.7);
	28.7–213.9	43.1–207.5
Falls	0.4 (1.7);	0.4 (1.4);
	0–52	0–50
MMSE	28.7 (1.8);	28.8 (1.8);
	0–30	0–30
Age (years)	61.7 (8.3);	61.5 (8.1);
	50–93	50–90
Anxiety	5.4 (3.6);	5.4 (3.5);
	0–20	0–20
Depression	5.6 (6.9);	5.4 (6.8);
	0–53	0–53
**Ordinal/Nominal Variable**	**Cohort 1 (Wave 1) Frequency (%)**	**Cohort 2 (Merged Wave 1–3) Frequency (%)**
Female	54.6	54.6
Education level		
- primary/none	21.2	19.5
- secondary	41.9	41.5
- third/higher	36.9	38.9
Antihypertensives	13.3	32.4
Diabetes	6.2	6.1
Smoker		
- never	45.9	46.7
- past	39.3	39.6
- current	14.9	13.6
Drinking problem	12.8(9.1 *)	13.2(7.6 *)
IPAQ		
- low	27.5	26.9
- medium	35.9	36.0
- high	35.7	36.2

* Dummy group of participants who answered “Don’t know” to the question “Do you have a drinking problem?”.

**Table 2:** Comparison of the OR and corresponding 95% C.I. of *bad performances*, *total mistakes,* and *mistakes in good performances* for the prediction of *TUG decline* in the binary logistic regression models.

TUG Decline									
	Bad Performances			Total Mistakes			Mistakes in Good Performances		
	OR	95% C.I.	*p*	OR	95% C.I.	*p*	OR	95% C.I.	*p*
Model 1	1.388	1.256–1.533	<0.001	1.048	1.039–1.056	<0.001	1.060	1.049–1.072	<0.001
Model 2	1.247	1.127–1.379	<0.001	1.042	1.030–1.053	<0.001	1.054	1.038–1.069	<0.001
Model 3	1.207	1.081–1.388	<0.001	1.027	1.015–1.040	<0.001	1.029	1.012–1.046	<0.001
Model 4	1.287	1.137–1.456	<0.001	1.029	1.015–1.043	<0.001	1.026	1.008–1.045	0.005
Model 4a	1.305	1.130–1.508	<0.001	1.030	1.015–1.047	<0.001	1.027	1.007–1.047	0.008

Models for each main predictor, i.e., *bad performances*, *total mistakes*, or *mistakes in good performances*: model 1, with just the main predictor; model 2, adjusted with mean RT and SD RT; model 3, which was model 2 with the addition of age, sex, and education level; model 4, the fully adjusted regression model, considering also the other covariates mentioned in Section 2.1.5 (anxiety, depression, hypertensives, diabetes, smoking, alcohol, and IPAQ); and model 4a, which was model 4 adjusted by UGS at baseline (wave 1). The odds ratio (OR) and corresponding 95% confidence interval (C.I.) give a measure of the influence of the predictor on the outcome; the *p*-value expresses the statistical significance of the predictor in the model.

**Table 3:** Comparison of the OR and corresponding 95% C.I. of *bad performances*, *total mistakes,* and *mistakes in good performances* for the prediction of *new fallers* in the binary logistic regression models.

New Fallers									
	Bad Performances			Total Mistakes			Mistakes in Good Performances		
	OR	95% C.I.	*p*	OR	95% C.I.	*p*	OR	95% C.I.	*p*
Model 1	1.114	1.040–1.194	0.002	1.014	1.007–1.021	<0.001	1.016	1.007–1.026	<0.001
Model 2	1.090	1.013–1.173	0.021	1.012	1.003–1.022	0.009	1.011	0.998–1.025	0.095
Model 3	1.076	0.998–1.160	0.057	1.008	0.998–1.018	0.123	1.003	0.990–1.017	0.646
Model 4	1.114	1.026–1.211	0.011	1.008	0.998–1.019	0.131	0.999	0.984–1.014	0.914
Model 4a	1.110	1.021–1.207	0.014	1.008	0.997–1.019	0.159	0.999	0.983–1.014	0.855

Models for each main predictor, i.e., *bad performances*, *total mistakes*, or *mistakes in good performances*: model 1, with just the main predictor; model 2, adjusted with mean RT and SD RT; model 3, which was model 2 with the addition of age, sex, and education level; model 4, the fully adjusted regression model, considering also all the other covariates mentioned in Section 2.1.5 (anxiety, depression, hypertensives, diabetes, smoking, alcohol, and IPAQ); and model 4a, which was model 4 adjusted by UGS at baseline (wave 1). The odds ratio (OR) and corresponding 95% confidence interval (C.I.) give a measure of the influence of the predictor on the outcome; the *p-*value expresses the statistical significance of the predictor in the model.

**Table 4:** Comparison of the OR and corresponding 95% C.I. of *bad performances*, *total mistakes,* and *mistakes in good performances* for the prediction of *MMSE decline* in the binary logistic regression models.

MMSE Decline									
	Bad Performances			Total Mistakes			Mistakes in Good Performances		
	OR	95% C.I.	*p*	OR	95% C.I.	*p*	OR	95% C.I.	*p*
Model 1	1.120	1.037–1.208	0.004	1.019	1.012–1.027	<0.001	1.026	1.016–1.036	<0.001
Model 2	1.067	0.981–1.159	0.129	1.014	1.004–1.024	0.004	1.019	1.005–1.033	0.006
Model 3	1.030	0.944–1.124	0.503	1.007	0.997–1.017	0.186	1.009	0.995–1.023	0.219
Model 4	1.067	0.969–1.174	0.187	1.010	0.999–1.021	0.082	1.011	0.995–1.026	0.178
Model 4a	1.063	0.965–1.170	0.216	1.010	0.998–1.021	0.090	1.011	0.995–1.027	0.180

Models for each main predictor, i.e., *bad performances*, *total mistakes*, or *mistakes in good performances*: model 1, with just the main predictor; model 2, adjusted with mean RT and SD RT; model 3, which was model 2 with the addition of age, sex, and education level; model 4, the fully adjusted regression model, considering also all the other covariates mentioned in Section 2.1.5 (anxiety, depression, hypertensives, diabetes, smoking, alcohol, and IPAQ); and model 4a, which was model 4 adjusted by UGS at baseline (wave 1). The odds ratio (OR) and corresponding 95% confidence interval (C.I.) give a measure of the influence of the predictor on the outcome; the *p*-value expresses the statistical significance of the predictor in the model.

## Data Availability

The datasets generated during and/or analysed during the current study are not publicly available due to data protection regulations, but are accessible at TILDA on reasonable request. The procedures to gain access to TILDA data are specified at https://tilda.tcd.ie/data/accessing-data/ (accessed on 14 January 2021).

## Appendix A

### SART RT Representation

An arithmetic average ${\bar{RT}}^{\nu }$ was computed for each trial ν, where ν = 1, …, 23, and considering non-null values only (i.e., excluding null values corresponding to the appearance of digit 3 on the screen). Therefore, we only considered the times when the participant correctly pressed the key, according to

(A1) $${\bar{RT}}^{\nu }=\frac{1}{M_{\nu }}\sum_{i=1}^{M_{\nu }}RT_{i}{}^{\nu },$$

where $M_{\nu }\in \left\{1,\ldots ,9\right\}$ is the number of times that the participant correctly pressed the key in the trial ν, or correctly did not press the key.

SART performance is known to be influenced by age [44], and therefore we recorded the age of participants and ordered the visualisations by increasing age as a continuous variable.

The ${\bar{RT}}^{\nu }\forall \nu \in \left\{1,\ldots ,23\right\}$ are plotted in a cloud plot (Figure 1) where the coordinates of each spot were given by the participant index in the age-sorted ascending order (x-coordinate) and by the ${\bar{RT}}^{\nu }$ for each trial ν, having then a total of 23 spots for each participant at the same point on the *x*-axis. The *x*-axis did not linearly follow the age variable, but it presented the age-sorted participants sequentially and equally spaced. For ease of interpretation, ticks were created to indicate a 5-year age change. Trials where the participant did not press the key at any time and/or some RT were missing did not have the computed average ${\bar{RT}}^{\nu }$ and were, thus, not plotted in the graph. The size of each spot $s_{\nu }\forall \nu \in \left\{1,\ldots ,23\right\}$ for each participant was given by

(A2) $$s_{\nu }=r_{\nu }{}^{-1},\;r_{\nu }=\frac{M_{\nu }}{9},$$

where $r_{\nu }$ is the ratio of the number of times that the key was correctly pressed (or not pressed) over the maximum possible number of correct pressing actions in the trial ν, i.e., 9. Therefore, bigger spots correspond to trials with higher number of mistakes. Moreover, the spots are colour-coded based on their size, i.e., the number of mistakes in the corresponding trial, going from light brown (0 mistakes) to black for the maximum number of mistakes (8 mistakes since in the case of 9 mistakes it is not possible to compute the mean RT and then assign a y-coordinate to the spot). Spots with size larger than 2 SD from the mean size $\bar{s}$ calculated across all the trials and all the participants (excluding missing data and trials with 0 correct actions), namely when the number of correct actions for the trial ν for the *i*-th participant was

(A3) $$M_{\nu ,i}=\frac{9}{\bar{s}+2SD}$$

were highlighted by white edges in the graph and labelled as “big spots”. The size $s_{\nu ,i}$ of each spot was scaled by a factor of 4 for clearer representation in the graph.

**Table A1:** Results for the fully adjusted per covariates binary logistic regression model 4 considering *bad performances* as potential predictor and having *TUG decline* as outcome.

TUG Decline			
Independent Variable	OR	95% C.I.	*p-*Value
Bad performances	1.287	1.137–1.456	<0.001
SART mean RT	1.001	0.999–1.002	0.340
SART SD RT	1.003	1.000–1.006	0.080
Age	1.136	1.115–1.159	<0.001
Females	1.171	0.863–1.590	0.311
Education level			
- primary/none	[ref]		
- secondary	0.930	0.646–1.337	0.695
- third/higher	0.985	0.682–1.423	0.936
Anxiety	1.030	0.983–1.081	0.217
Depression	1.014	0.990–1.038	0.249
Antihypertensives	1.942	1.451–2.600	<0.001
Diabetes	1.678	1.051–2.680	0.030
Smoker			
- never	[ref]		
- past	1.012	0.743–1.380	0.938
- current	1.790	1.147–2.795	0.010
Drinking problem			
- “No”	[ref]		
- “Don’t know”	0.676	0.197–2.313	0.532
- “Yes”	0.989	0.626–1.564	0.964
IPAQ			
- low	[ref]		
- medium	0.802	0.572–1.125	0.201
- high	0.684	0.474–0.986	0.042

**Table A2:** Results for the fully adjusted per covariates binary logistic regression model 4a considering *bad performances* as potential predictor and having *TUG decline* as outcome.

TUG Decline			
Independent Variable	OR	95% C.I.	*p*-Value
Bad performances	1.305	1.130–1.508	<0.001
SART mean RT	1.000	0.999–1.002	0.791
SART SD RT	1.003	1.000–1.007	0.074
Age	1.102	1.080–1.125	<0.001
Females	0.891	0.646–1.231	0.485
Education level			
- primary/none	[ref]		
- secondary	1.075	0.732–1.579	0.711
- third/higher	1.246	0.843–1.841	0.270
Anxiety	1.023	0.973–1.075	0.382
Depression	1.007	0.982–1.033	0.570
Antihypertensives	1.672	1.230–2.273	0.001
Diabetes	1.348	0.818–2.220	0.241
Smoker			
- never	[ref]		
- past	0.951	0.685–1.319	0.762
- current	1.581	0.989–2.529	0.056
Drinking problem			
- “No”	[ref]		
- “Don’t know”	0.660	0.178–2.443	0.534
- “Yes”	0.907	0.562–1.463	0.689
UGS at baseline	0.938	0.928–0.948	<0.001
IPAQ			
- low	[ref]		
- medium	0.959	0.671–1.370	0.819
- high	0.815	0.554–1.199	0.299

**Table A3:** Results for the fully adjusted per covariates binary logistic regression model 4 considering *bad performances* as potential predictor and having *UGS decline* as outcome.

UGS Decline			
Independent Variable	OR	95% C.I.	*p*-Value
Bad performances	1.057	0.903–1.237	0.492
SART mean RT	1.001	0.999–1.003	0.301
SART SD RT	1.003	0.998–1.008	0.241
Age	1.093	1.064–1.122	<0.001
Females	1.178	0.752–1.845	0.474
Education level			
- primary/none	[ref]		
- secondary	1.088	0.633–1.870	0.761
- third/higher	1.078	0.615–1.890	0.792
Anxiety	0.999	0.932–1.072	0.986
Depression	1.032	0.999–1.066	0.061
Antihypertensives	1.767	1.138–2.745	0.011
Diabetes	1.558	0.794–3.057	0.198
Smoker			
- never	[ref]		
- past	0.905	0.573–1.427	0.666
- current	1.351	0.694–2.628	0.376
Drinking problem			
- “No”	[ref]		
- “Don’t know”	1.849	0.537–6.371	0.330
- “Yes”	1.093	0.563–2.121	0.793
IPAQ			
- low	[ref]		
- medium	0.855	0.530–1.379	0.520
- high	0.657	0.382–1.130	0.129

**Table A4:** Results for the fully adjusted per covariates binary logistic regression model 4 considering *bad performances* as potential predictor and having *New faller* as outcome.

New Fallers			
Independent Variable	OR	95% C.I.	*p*-Value
Bad performances	1.114	1.026–1.211	0.011
SART mean RT	0.999	0.998–1.001	0.298
SART SD RT	1.001	0.999–1.004	0.319
Age	1.020	1.006–1.034	0.004
Females	1.237	0.997–1.536	0.053
Education level			
- primary/none	[ref]		
- secondary	1.033	0.779–1.370	0.821
- third/higher	1.065	0.799–1.420	0.667
Anxiety	1.002	0.969–1.036	0.917
Depression	1.014	0.998–1.031	0.092
Antihypertensives	1.184	0.947–1.481	0.139
Diabetes	1.111	0.741–1.668	0.610
Smoker			
- never	[ref]		
- past	1.200	0.579–2.489	0.624
- current	1.042	0.769–1.412	0.792
Drinking problem			
- “No”	[ref]		
- “Don’t know”	0.971	0.755–1.248	0.816
- “Yes”	0.905	0.697–1.176	0.457
IPAQ			
- low	[ref]		
- medium	1.002	0.969–1.036	0.917
- high	1.014	0.998–1.031	0.092

**Table A5:** Results for the fully adjusted per covariates binary logistic regression model 4a considering *bad performances* as potential predictor and having *New faller* as outcome.

New Fallers			
Independent Variable	OR	95% C.I.	*p*-Value
Bad performances	1.110	1.021–1.207	0.014
SART mean RT	0.999	0.998–1.001	0.278
SART SD RT	1.002	0.999–1.004	0.264
Age	1.019	1.004–1.034	0.011
Females	1.232	0.990–1.534	0.061
Education level			
- primary/none	[ref]		
- secondary	1.020	0.768–1.354	0.892
- third/higher	1.043	0.781–1.393	0.775
Anxiety	1.004	0.971–1.038	0.826
Depression	1.014	0.998–1.031	0.089
Antihypertensives	1.174	0.935–1.474	0.166
Diabetes	1.113	0.736–1.682	0.613
Smoker			
- never	[ref]		
- past	1.148	0.920–1.434	0.222
- current	1.071	0.768–1.493	0.688
Drinking problem			
- “No”	[ref]		
- “Don’t know”	1.206	0.581–2.501	0.615
- “Yes”	1.033	0.760–1.403	0.837
UGS at baseline	0.999	0.994–1.005	0.851
IPAQ			
- low	[ref]		
- medium	0.960	0.745–1.237	0.751
- high	0.892	0.685–1.163	0.399

**Table A6:** Comparison of the OR and corresponding 95% C.I. of *bad performances*, *total mistakes* and *mistakes in good performances* for the prediction of *UGS decline* in the binary logistic regression models.

UGS Decline									
	Bad Performances			Total Mistakes			Mistakes in Good Performances		
	OR	95% C.I.	*p*	OR	95% C.I.	*p*	OR	95% C.I.	*p*
Model 1	1.232	1.102–1.377	<0.001	1.036	1.024–1.047	<0.001	1.049	1.033–1.066	<0.001
Model 2	1.126	0.993–1.277	0.064	1.025	1.010–1.041	0.002	1.036	1.014–1.060	0.001
Model 3	1.058	0.917–1.222	0.438	1.010	0.993–1.028	0.242	1.016	0.992–1.040	0.205
Model 4	1.057	0.903–1.237	0.492	1.013	0.994–1.033	0.180	1.022	0.995–1.049	0.105

Models for each main predictor, i.e., *bad performances*, *total mistakes*, or *mistakes in good performances*: model 1, with just the predictor; model 2, adjusted with mean RT and SD RT; model 3, which was model 2 with the addition of age, sex, and education level; model 4, the fully adjusted regression model, considering also the other covariates mentioned in Section 2.1.5 (anxiety, depression, hypertensives, diabetes, smoking, alcohol, and IPAQ). The odds ratio (OR) and corresponding 95% confidence interval (C.I.) give a measure of the influence of the predictor on the outcome; the *p-*value expresses the statistical significance of the predictor in the model.

**Table A7:** Results for the fully adjusted per covariates binary logistic regression model 4 considering *bad performances* as potential predictor and having *MMSE decline* as outcome.

MMSE Decline			
Independent Variable	OR	95% C.I.	*p*-Value
Bad performances	1.067	0.969–1.174	0.187
SART mean RT	1.000	0.999–1.002	0.495
SART SD RT	1.001	0.999–1.004	0.334
Age	1.044	1.029–1.059	<0.001
Females	0.819	0.651–1.031	0.090
Education level			
- primary/none	[ref]		
- secondary	0.797	0.600–1.058	0.117
- third/higher	0.747	0.557–1.001	0.051
Anxiety	1.012	0.976–1.049	0.512
Depression	1.011	0.993–1.029	0.239
Antihypertensives	1.104	0.870–1.402	0.416
Diabetes	0.759	0.473–1.217	0.252
Smoker			
- never	[ref]		
- past	1.075	0.844–1.368	0.558
- current	1.523	1.092–2.123	0.013
Drinking problem			
- “No”	[ref]		
- “Don’t know”	0.717	0.280–1.832	0.487
- “Yes”	0.925	0.661–1.293	0.647
IPAQ			
- low	[ref]		
- medium	1.143	0.863–1.514	0.350
- high	1.192	0.895–1.588	0.230

**Table A8:** Results for the fully adjusted per covariates binary logistic regression model 4a considering *bad performances* as potential predictor and having *MMSE decline* as outcome.

MMSE Decline			
Independent Variable	OR	95% C.I.	*p*-Value
Bad performances	1.063	0.965–1.170	0.216
SART mean RT	1.000	0.999–1.002	0.559
SART SD RT	1.001	0.998–1.004	0.375
Age	1.041	1.025–1.057	<0.001
Females	0.816	0.647–1.029	0.086
Education level			
- primary/none	[ref]		
- secondary	0.792	0.596–1.053	0.108
- third/higher	0.738	0.549–0.991	0.043
Anxiety	1.012	0.976–1.049	0.535
Depression	1.011	0.993–1.029	0.233
Antihypertensives	1.093	0.858–1.392	0.471
Diabetes	0.738	0.455–1.197	0.218
Smoker			
- never	[ref]		
- past	1.070	0.840–1.363	0.584
- current	1.491	1.066–2.085	0.020
Drinking problem			
- “No”	[ref]		
- “Don’t know”	0.714	0.279–1.825	0.482
- “Yes”	0.911	0.650–1.278	0.590
UGS at baseline	0.998	0.991–1.004	0.436
IPAQ			
- low	[ref]		
- medium	1.125	0.848–1.494	0.414
- high	1.203	0.902–1.606	0.209
